# Supplementary material for: A connectome of a learning and memory center in the adult Drosophila brain
Source: eLife. 2017 Jul 18;6:e26975. doi: 10.7554/eLife.26975 (PMC5550281; doi:10.7554/eLife.26975)
Supplement: Figure 6—source data 1. — Numbers of synapses detected between different circuit elements corresponding to the graphical display in Figure 6. Left column indicates the compartment where the connections were observed, and the next column shows cell types presynaptic to the cell types shown in the top row. In α3, PPL refers specifically to two PPL1-α3neurons, in α2 it is the two PPL1-α′2α2 cells and in α1 it is 16 PAM-α1 neurons. The MBONs do not make any output synapses within the lobes and so are omitted from the rows of the matrix. Red shading indicates synaptic connections newly identified in this study. DOI: http://dx.doi.org/10.7554/eLife.26975.022 [file elife-26975-fig6-data1.docx]

|  | To  From | KC α/βc(i) | KC α/βc(o) | KC α/βs | KC α/βp | PPL | PAM | MBON- α3 | MBON- α2sc | MBON- α2sp | MBON- α2p3p | MBON- α1 | APL | DPM |
| --- | --- | --- | --- | --- | --- | --- | --- | --- | --- | --- | --- | --- | --- | --- |
| α3 | KC α/βc(i) | 6787 | 1220 | 367 | 0 | 1439 | 0 | 5550 | 63 | 0 | 0 | 0 | 1004 | 348 |
| α2 | KC α/βc(i) | 4169 | 510 | 72 | 0 | 556 | 15 | 308 | 2560 | 334 | 7 | 162 | 1004 | 341 |
| α1 | KC α/βc(i) | 7039 | 774 | 126 | 0 | 2 | 637 | 0 | 4 | 0 | 0 | 3706 | 1003 | 146 |
| α3 | KC α/βc(o) | 1108 | 1991 | 1338 | 0 | 848 | 0 | 3317 | 52 | 0 | 0 | 0 | 436 | 384 |
| α2 | KC α/βc(o) | 532 | 1472 | 652 | 0 | 314 | 23 | 72 | 1753 | 456 | 29 | 88 | 513 | 357 |
| α1 | KC α/βc(o) | 783 | 1812 | 1055 | 0 | 0 | 397 | 0 | 0 | 9 | 0 | 2098 | 454 | 263 |
| α3 | KC α/βs | 365 | 1428 | 19922 | 373 | 2552 | 0 | 15425 | 362 | 40 | 13 | 0 | 1459 | 2123 |
| α2 | KC α/βs | 75 | 717 | 9403 | 220 | 560 | 46 | 31 | 6420 | 1984 | 389 | 250 | 1374 | 1237 |
| α1 | KC α/βs | 134 | 1099 | 13346 | 413 | 1 | 1588 | 0 | 0 | 12 | 0 | 10111 | 1136 | 1109 |
| α3 | KC α/βp | 0 | 0 | 293 | 1806 | 184 | 0 | 1196 | 5 | 59 | 158 | 0 | 326 | 344 |
| α2 | KC α/βp | 0 | 0 | 138 | 1378 | 178 | 0 | 0 | 62 | 633 | 1407 | 0 | 228 | 315 |
| α1 | KC α/βp | 0 | 2 | 338 | 2380 | 3 | 337 | 0 | 0 | 2 | 0 | 2155 | 142 | 230 |
| α3 | PPL | 929 | 461 | 1346 | 253 | 2 | 0 | 906 | 2 | 1 | 2 | 0 | 23 | 54 |
| α2 | PPL | 223 | 184 | 685 | 359 | 0 | 0 | 2 | 246 | 89 | 36 | 0 | 82 | 100 |
| α1 | PPL | 0 | 0 | 1 | 4 | 0 | 0 | 0 | 0 | 1 | 0 | 0 | 0 | 0 |
| α3 | PAM | 0 | 0 | 0 | 0 | 0 | 0 | 0 | 0 | 0 | 0 | 0 | 0 | 0 |
| α2 | PAM | 9 | 9 | 31 | 0 | 0 | 0 | 0 | 0 | 0 | 0 | 22 | 0 | 0 |
| α1 | PAM | 300 | 271 | 1011 | 364 | 0 | 24 | 0 | 0 | 0 | 0 | 1422 | 21 | 22 |
| α3 | APL | 236 | 140 | 950 | 115 | 0 | 0 | 0 | 0 | 0 | 0 | 0 | 0 | 11 |
| α2 | APL | 178 | 132 | 850 | 107 | 0 | 0 | 0 | 0 | 0 | 0 | 1 | 0 | 12 |
| α1 | APL | 158 | 160 | 990 | 96 | 0 | 0 | 0 | 0 | 0 | 0 | 62 | 0 | 16 |
| α3 | DPM | 123 | 116 | 577 | 101 | 43 | 0 | 8 | 1 | 0 | 1 | 0 | 86 | 0 |
| α2 | DPM | 103 | 109 | 357 | 70 | 79 | 7 | 0 | 20 | 7 | 5 | 7 | 56 | 0 |
| α1 | DPM | 45 | 58 | 245 | 62 | 0 | 73 | 0 | 0 | 0 | 0 | 238 | 24 | 0 |

Figure 6 – source data 1
